# Optimal teaching strategy in periodic impulsive knowledge dissemination system

**DOI:** 10.1371/journal.pone.0178024

**Published:** 2017-06-30

**Authors:** Dan-Qing Liu, Zhen-Qiang Wu, Yu-Xin Wang, Qiang Guo, Jian-Guo Liu

**Affiliations:** 1 School of Computer Science, Shaanxi Normal University, Xi’an, PR China; 2 School of Computer, Qinghai Normal University, Xining, PR China; 3 Key Laboratory of Modern Teaching Technology, Ministry of Education, Xi’an, PR China; 4 Sichuan Institute for Educational Research, Chengdu, PR China; 5 Research Center of Complex Systems Science, University of Shanghai for Science and Technology, Shanghai, PR China; 6 Data Science and Cloud Service Research Center, Shanghai University of Finance and Economics, Shanghai, PR China; Beihang University, CHINA

## Abstract

Accurately describing the knowledge dissemination process is significant to enhance the performance of personalized education. In this study, considering the effect of periodic teaching activities on the learning process, we propose a periodic impulsive knowledge dissemination system to regenerate the knowledge dissemination process. Meanwhile, we put forward learning effectiveness which is an outcome of a trade-off between the benefits and costs raised by knowledge dissemination as objective function. Further, we investigate the optimal teaching strategy which can maximize learning effectiveness, to obtain the optimal effect of knowledge dissemination affected by the teaching activities. We solve this dynamic optimization problem by optimal control theory and get the optimization system. At last we numerically solve this system in several practical examples to make the conclusions intuitive and specific. The optimal teaching strategy proposed in this paper can be applied widely in the optimization problem of personal education and beneficial for enhancing the effect of knowledge dissemination.

## Introduction

Personalized education has attracted lots of attention for enhancing the performance of teaching and learning, which could set specific educational objectives, teaching plans, guidance programs, and executive management system according to the performance of a learner [1–4]. The effect of knowledge dissemination in personalized education is closely related to how we describe the knowledge dissemination process [5, 6]. So far, knowledge dissemination models mainly focus on the learning rules [7], the memory retention [8, 9] and forgetting mechanisms [10, 11].

Hicklin [12] proposed a theoretical model taking into account individual learning in a given ideal learning situation. He envisaged that learning resulted from a dynamic equilibrium between information acquisition and loss, in which the rate of information gain was affected only by the individual’s aptitude for learning and the probability of information being forgotten. Anderson [13] developed an experienced mathematical model by considering student’s intelligence, abstract stimulus information and knowledge density of a student. This model focused on the effect of knowledge characteristics on knowledge growth, and disregarded individual internal and environmental factors. Benfenati [14] investigated the cellular and molecular mechanisms that contribute to various forms of memories, including short- and long-term memories, as well as unconscious and conscious memories. Other important models of forgetting process are the composite holographic associative recall model proposed by Metcalfe [15] and Chappell [16], the matrix model proposed by Humphreys [17] and the multiple-trace simulation model proposed by Hintzman [18]. Taking into account the brain switching process, Roy [19] built a dynamical model which given a systematic mathematical description for both the learning and forgetting processes. This model could map the knowledge dissemination process in self-regulated learning [20].

Since the growth of knowledge stock of a learner not only depends on the individual learning and forgetting abilities, but also depends on the teacher guidance, our attention naturally focuses on seeing how knowledge grows and changes after the teaching activities. In practice, evident and major changes of knowledge stock caused by such activities can be assumed as subjected to impulsive perturbations in short-term. Impulsive differential equations exactly provide the natural description for such notable changes in quantity in the short run [21]. Therefore, we can establish a impulsive knowledge dissemination system to map the knowledge dissemination process with impulsive perturbations.

Generally speaking, personalized education always has a strong sense of purpose. On the one hand, learners eagerly hope that knowledge can bring benefits, such as improving self-efficacy or increasing academic and economic profits [22]. On the other hand, the teaching activities typically consume considerable manpower, material, and financial resources that require payment. These two aspects of knowledge dissemination system exhibit a relationship of mutual restriction. Learning effectiveness is an outcome of a trade-off between the benefits and costs. Thus, we can propose learning effectiveness as objective function, which exactly reflects how well a knowledge dissemination system performs [23].

In this paper, we devote to investigate the optimal teaching strategy which can maximize learning effectiveness, to obtain the optimal effect of knowledge dissemination affected by the teaching activities. That is to say, we need to expense minimum costs in exchange for maximum benefits. It is an optimization problem of teaching strategy in knowledge dissemination. Inspired by the studies on the optimization problems of management objectives in the application areas of impulsive differential equation [24, 25], we generalize the common method, such as optimal control theory [26], to solve this extremum problem presented in our study.

## Modeling

### 2.1 Construction of the Roy model

Considering the influences of individual internal factors on self-regulated learning, Roy [19] established a systematical ordinary differential equation for the learning process, which is described briefly in this section. He used *X*(*t*) to represent the amount of knowledge already stored in the brain at a current time. From the common experiences of people, the rate of knowledge storage (*R*_*S*_) can be calculated simply by subtracting the rate of knowledge loss (*R*_*L*_) from the rate of knowledge entry (*R*_*E*_).

For the memory retention mechanisms, we know the rate of knowledge entry should be relevant to the ability like grasping power, concentration, intelligence and urgency of learning etc. It is a common experience that as the accumulated knowledge increases in the brain, the rate of knowledge entry must decrease due to brain fatigue or some mental stress [19]. Similarity, for the memory forgetting mechanisms, experience tells us that the rate of knowledge loss become increasingly rapidly when storing more and more knowledge, possibly owing to the limitation of retention ability and the stress caused by the load of already accumulated knowledge [19].

Then a simple mathematical formula in the following form can be obtained: where *C* denotes the maximum storage capacity of a subject; *C*_1_ and *C*_2_ denote the capability of a learner to absorb knowledge and retain memory, respectively. Parameters *α* and *β* are positive quantities, which may be called the brain fatigue index and the stress endurance index, respectively

$$\begin{array}{c} R_{S}=\frac{dX}{dt}=R_{E}-R_{L}=s\left(t\right)C_{1}{\left(1-\frac{X}{C}\right)}^{\alpha }-\frac{{\left(X/C\right)}^{\beta }}{C_{2}}. \end{array}$$ (1)

Here, *s*(*t*) is a time dependent switching function that ranges from 0 to 1. It can finely characterize the states of knowledge entering into brain. The function of *s*(*t*) can be approximated by the two tan-hyperbolic functions given below for exactly simulating the two main learning scenarios, where *T*_*m*_ is the duration during which a learner maintains conscious learning efforts without any break

$$\begin{array}{c} s\left(t\right)=1-\frac{1}{2}\left(\tanh k\left(t-T_{m}\right)+1\right), \end{array}$$ (2)

$$\begin{array}{c} s\left(t\right)=\frac{1}{2}\left(\tanh k\left(\sin\frac{\pi t}{T_{m}}\right)+1\right). \end{array}$$ (3)

**Fig 1:**
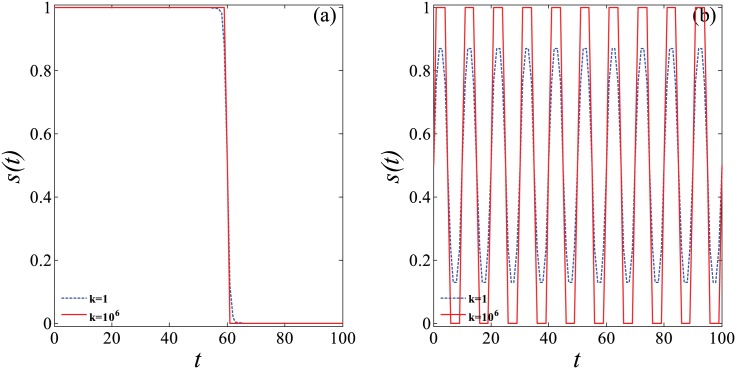
(Color online) Graphical representation of Eqs (2) and (3) for parametric variations. (a) *s*(*t*) follows Eq (2), *T*_*m*_ = 60; (b) *s*(*t*) follows Eq (3), *T*_*m*_ = 5.

It is evident that, for a sufficiently large positive *k* value, the function of *s*(*t*) behaves similar to the values of the alternating 0 and 1. As shown in Fig 1(a) in which *s*(*t*) adopts Eq (2), when *s*(*t*) approximately equals 1, knowledge enters coexisting with loss in the continuous learning process. On the opposite, when *s*(*t*) nearly equals 0 from *t* = *T*_*m*_ onwards, only the forgetting mechanism remains. Hence, Eq (2) can be used to describe the scenario in which the learning activities sustain throughout the entire semester and relax during the vacation. By contrast, Eq (3) always presents the periodic variation (by a cycle of 2*T*_*m*_), as shown in Fig 1(b). The influences of *s*(*t*) on the rate of knowledge stock storing in the brain are the same as aforementioned. Obviously, the Eq (3) is used to simulate a scene, where the learning activities are scheduled periodically, and thus active learning and forgetting alternately dominate the learning process periodically.

The Model (1) can be rescaled to nondimensional form by using the substitutions *x* = *X*/*C*, $\eta_{1}=C_{1}/C_{1}^{max}$ and $\eta_{2}=C_{2}/C_{2}^{max}$. Here, *η*_1_ and *η*_2_ are the merit index and the memory index to quantify intelligence quotient and memory retention ability of a learner relative to the best learner, respectively. The parameters $C_{1}^{max}$ and $C_{2}^{max}$ are the values of *C*_1_ and *C*_2_ for the best possible learner, and generally assumed as 1 for calculation convenience. Hence, Model (1) can be rewritten as

$$\begin{array}{c} \frac{dx}{dt}=\frac{s\left(t\right)\eta_{1}}{C}{\left(1-x\right)}^{\alpha }-\frac{x^{\beta }}{C\eta_{2}}. \end{array}$$ (4)

**Fig 2:**
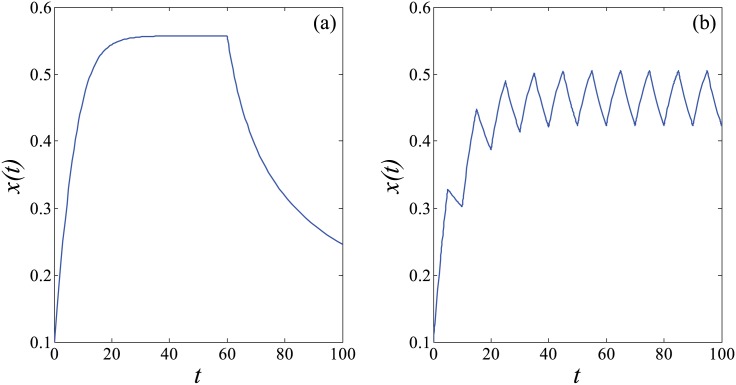
*X*-*t* variation in self-regulated learning. We set parameters to *η*_1_ = 0.6, *η*_2_ = 0.6, *α* = 0.9, *β* = 3, *C* = 10, *k* = 10^6^, *x*(0) = 0.1 in Eq (4). (a) *s*(*t*) follows Eq (2), *T*_*m*_ = 60; (b) *s*(*t*) follows Eq (3), *T*_*m*_ = 5.

Then the variation of knowledge stock in the two main learning scenarios over time can be depicted through numerical simulation in Fig 2.

### 2.2 Construction of periodic impulsive system

Compared with self-regulated learning in long periods, teaching activity with a relatively short term can be seen as an instantaneous process. Teacher is generally considered as a highly learned individual who shares his or her knowledge with learners. We assume that teachers have sufficient teaching skills and extensive subject knowledge to enable learners to master the relevant knowledge well within a short period. Considering the influences of such environmental factors (e.g., teaching activities) on self-regulated learning, an impulsive knowledge dissemination system can be used to describe the variation of knowledge stock in this situation as follows

$$\begin{array}{c} \left\{ \begin{array}{cc} \frac{dx}{dt}=\frac{s\left(t\right)\eta_{1}}{C}{\left(1-x\right)}^{\alpha }-\frac{x^{\beta }}{C\eta_{2}}, & t\neq t_{i},\\ x\left(t_{i}^{+}\right)=x\left(t_{i}\right)+E_{i}\left(1-x\left(t_{i}\right)\right), & t=t_{i}. \end{array}\right. \end{array}$$ (5)

**Fig 3:**
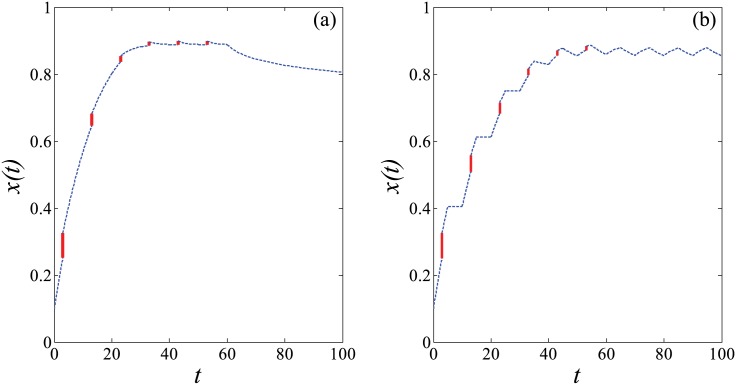
(Color online) Variation of *X* with *t* in periodic impulsive system. We set parameters to *η*_1_ = 0.6, *η*_2_ = 0.6, *α* = 0.9, *β* = 3, *C* = 10, *k* = 10^6^, *x*(0) = 0.1, with one impulse effect (*E*_1_ = 0.1) at only one fixed moment (*t*_1_ = 3) per period (*T* = 10) for six periods (*N* = 6) in Eq (5). (a) *s*(*t*) follows Eq (2), *T*_*m*_ = 60; (b) *s*(*t*) follows Eq (3), *T*_*m*_ = 5. The blue dotted curves display the growth of knowledge stock in self-regulated learning over time. While the red solid lines show the rapidly increasing amount of knowledge within a short time after the teacher guidance.

The second equation of System (5) quantitatively describes the significant change of knowledge stock after the transitory teaching activities, *x*(*t*_*i*_) is the amount of knowledge already stored in the brain before guidance, and 1 − *x*(*t*_*i*_) is the remaining knowledge required to be learned or mastered at time *t* = *t*_*i*_. The teaching effort, *E*_*i*_(0 ≤ *E*_*i*_ < 1), represents the percentages of the residual knowledge that need to be taught according to the current learning performance, which is restricted to knowledge absorptive capacity of a learner. Apparently, we can rapidly raise knowledge stock from *x*(*t*_*i*_) to $x(t_{i}^{+})$ with a scale of *E*_*i*_ in a teaching activity at time *t* = *t*_*i*_. For the two different learning scenarios distinguished with Eqs (2) and (3), we can simulate how the amount of knowledge changes on account of imposing the periodic impulsive teaching effects. These two systems exactly behave as shown in Fig 3, with one impulse effect (*E*_1_ = 0.1) at only one fixed moment (*t*_1_ = 3) per period (*T* = 10) for six periods (*N* = 6).

Studying the periodic system is important and reasonable since the learning process is always subjected to evident periodic fluctuations [27]. For example, teaching activities typically occur at fixed moments every week or in regular pulses throughout the entire semester. The learning or memorizing abilities of a learner exhibit periodic changes because of such periodic fluctuations as well. Without loss of generality, a common assumption for System (5) is that all the functions are periodic with the same period. So we assume that *η*_1_(*t*) and *η*_2_(*t*) are the same continuous *T*–period functions with *s*(*t*) (given that Eq (2) is non-periodic, we only consider Eq (3) in the follow-up research). Besides, we hypothesize that *q* times impulse effects occur at time {*t* = *t*_*i*_, *i* = 1, 2, ⋯, *q*} per period, namely, there exists a positive integer *q* that satisfies *t*_*i*+*q*_ = *t*_*i*_+*T* and *E*_*i* + *q*_ = *E*_*i*_ for all *i* ∈ *N*^+^. We mainly study the optimal control problem under the periodic conditions. That is, the solutions of System (5) are also required to be periodic, i.e.

$$\begin{array}{c} x(t)=x(t+T). \end{array}$$ (6)

Here, *x*(*t*) is required to be continuously differentiable at *t* ≠ *t*_*i*_ and left continuous at *t* = *t*_*i*_. Moreover, $x\left(t_{i}^{+}\right)=\lim_{h\to 0^{+}}x\left(t_{i}+h\right)$ must exist. Consequently, Systems (5) and (6) can constitute the following *T*–periodic impulsive knowledge dissemination system

$$\begin{array}{c} \left\{ \begin{array}{cc} \frac{dx}{dt}=\frac{s\left(t\right)\eta_{1}\left(t\right)}{C}{\left(1-x(t)\right)}^{\alpha }-\frac{x^{\beta }\left(t\right)}{C\eta_{2}\left(t\right)}, & t\neq t_{i},\\ x\left(t_{i}^{+}\right)=x\left(t_{i}\right)+E_{i}\left(1-x\left(t_{i}\right)\right), & t=t_{i},\\ x(t)=x(t+T). \end{array}\right. \end{array}$$ (7)

### 2.3 Construction of dynamic optimization problem

This study aims to find the optimal teaching strategy for the knowledge dissemination system. Thus, we can select teaching efforts {*E*_*i*_, *i* = 1, 2, ⋯, *q*} as control variables (assuming *t*_*i*_, *i* = 1, 2, ⋯, *q* are fixed) and learning effectiveness as objective function. And the performance index function can be expressed as

$$\begin{array}{c} J\left(E\right)=\sum_{i=1}^{q}\left(P\left(1-x\left(t_{i}\right)\right)E_{i}-LE_{i}\right). \end{array}$$ (8)

In Eq (8), we use the positive constants *P* and *L* between 0-10 as indexes to represent the benefits and costs raised by per effort respectively. *x* = *x*(*t*) is the *T*–periodic and unique positive solution of System (7) under control variables {*E*_*i*_, *i* = 1, 2, ⋯, *q*}. $\sum_{i=1}^{q}P\left(1-x\left(t_{i}\right)\right)E_{i}$ and $\sum_{i=1}^{q}LE_{i}$ represent the total benefits and costs per period, respectively. Then learning effectiveness *J* can be obtained by the difference of the two aspects.

According to actual problem, we define the admissible set of System (7) as *S* = {*E*_*i*_∣*E*_*i*+*q*_ = *E*_*i*_, 0 ≤ *E*_*i*_ < 1, *i* = 1, 2, ⋯, *q*}. The optimal control rule is to maximize objective function when control variables are selected in the admissible set, which is a dynamic optimization problem of a function. Hence, this control problem can be described as

$$\begin{array}{c} \left\{ \begin{array}{cc} \max & J\left(E\right)=\sum_{i=1}^{q}\left(P\left(1-x\left(t_{i}\right)\right)E_{i}-LE_{i}\right),\\ \text{S.t.} & \Delta x^{\left(i\right)}=x\left(t_{i}^{+}\right)-x\left(t_{i}\right)=E_{i}\left(1-x\left(t_{i}\right)\right). \end{array}\right. \end{array}$$ (9)

If there exists an control strategy E* ∈ *S* satisfying the above optimal problem, then $\left\{E_{i}^{*},i=1,2,\cdots ,q\right\}$ is an optimal control sequence (called the optimal impulsive teaching strategy), and {x*(*t*_*i*_), *i* = 1, 2, ⋯, *q*} is the corresponding optimal trajectory (called the optimal knowledge stock level). All of them are also the optimal solutions of System (7). We settle this extremal problem by discrete time optimal control theory and generate the optimization system in the end, from which we can obtain these numerical optimal solutions.

## Methods

### 3.1 Existence of optimal strategy

In order to show the process of analysis and solution more intuitively and clearly, we just analyze the properties of the analytical solution of System (7) and successively illustrate the existence of the optimal impulsive teaching strategy when *α* = *β* = 1. We also can get the numerical optimal solutions by numerical simulation in other cases.

System (7) can be rewritten in the following form Eq (10), also known as the state equations

$$\begin{array}{c} \left\{ \begin{array}{cc} \frac{dx}{dt}=-\left(\frac{s\left(t\right)\eta_{1}\left(t\right)}{C}+\frac{1}{C\eta_{2}\left(t\right)}\right)x\left(t\right)+\frac{s\left(t\right)\eta_{1}\left(t\right)}{C}, & t\neq t_{i},\\ x\left(t_{i}^{+}\right)=\left(1-E_{i}\right)x\left(t_{i}\right)+E_{i}, & t=t_{i},\\ x(t)=x(t+T). \end{array}\right. \end{array}$$ (10)

We define

$$\begin{array}{c} F_{1}\left(t\right)=\frac{s\left(t\right)\eta_{1}\left(t\right)}{C}+\frac{1}{C\eta_{2}\left(t\right)},F_{2}\left(t\right)=\frac{s\left(t\right)\eta_{1}\left(t\right)}{C}. \end{array}$$ (11)

From *T*–periodicity of System (10), there exists *T* > 0 and *q* ∈ *N*^+^ satisfying Condition (12)

$$\begin{array}{c} \left\{ \begin{array}{cc} F_{1}\left(t+T\right)=F_{1}\left(t\right),F_{2}\left(t+T\right)=F_{2}\left(t\right), & (t\in R),\\ t_{i+q}=t_{i}+T,E_{i+q}=E_{i},0\leq E_{i}<1, & (i\in N^{+}),\\ F_{1}\left(t\right),F_{2}\left(t\right)\in PC\left(R,R\right),F_{2}\left(t\right)>0, & (t\in R). \end{array}\right. \end{array}$$ (12)

The unique solution of System (10) with positive initial value *x*_0_ = *x*(0) can be formulated as, for all *t* > 0

$$\begin{array}{ccc} x(t) & = & x\left(0\right)\prod_{0<t_{i}<t}\left(1-E_{i}\right)e^{-\int_{0}^{t}F_{1}\left(s\right)ds}+\sum_{0<t_{i}<t}\left(\prod_{t_{i}<t_{j}<t}\left(1-E_{j}\right)e^{-\int_{t_{i}}^{t}F_{1}\left(s\right)ds}\right)E_{i}\\ & & +\int_{0}^{t}\prod_{s<t_{i}<t}\left(1-E_{i}\right)e^{-\int_{s}^{t}F_{1}\left(\sigma \right)d\sigma }F_{2}\left(s\right)ds. \end{array}$$ (13)

In addition, we have *x*(0) = *x*(*T*) for *T*–periodic solution. Then we can obtain the following *x*(0) from Eq (13)

$$\begin{array}{ccc} x(0) & = & {\left(1-\prod_{i=1}^{q}\left(1-E_{i}\right)e^{-\int_{0}^{T}F_{1}\left(s\right)ds}\right)}^{-1}(\sum_{i=1}^{q}\left(\prod_{t_{i}<t_{j}<t}\left(1-E_{j}\right)e^{-\int_{t_{i}}^{T}F_{1}\left(s\right)ds}\right)E_{i}\\ & & +\int_{0}^{T}\prod_{s<t_{i}<t}\left(1-E_{i}\right)e^{-\int_{s}^{T}F_{1}\left(\sigma \right)d\sigma }F_{2}\left(s\right)ds). \end{array}$$ (14)

Substituting Eq (14) into Eq (13) can yield the explicit expressions of *T*–periodic solution, denoted as *x*^*T*^(*t*).

Give that *F*_1_(*t*) > 0, *F*_2_(*t*) > 0, and 0 ≤ *E*_*i*_ < 1, it is easy to prove that *x*^*T*^(*t*) is positive for all *t* ≥ 0, with positive initial value *x*(0). It is also uniformly bounded. Moreover, from Theorem of the existence and uniqueness of the periodic solution for linear impulsive differential system, we postulate that the Condition (15) holds

$$\begin{array}{c} \mu =\prod_{i=1}^{q}{\left(1-E_{i}\right)}^{-1}e^{\int_{0}^{T}F_{1}\left(t\right)dt}>1. \end{array}$$ (15)

Therefore, System (10) implies that *x*^*T*^(*t*) with positive initial value, which exists uniquely, is positive, uniformly bounded, and globally attracts all other positive solutions for all impulsive teaching efforts *E*_*i*_ ∈ *S*(*i* = 1, 2, ⋯, *q*).

Because of the properties above of *x*^*T*^(*t*), we can obtain

$$\begin{array}{c} \underset{E\in S}{\sup}J\left(E\right)<+\infty . \end{array}$$ (16)

Besides, *J*(*E*) continuously depends on *E*, and *S* is a closed set. Thus, there must exist an optimal control E* ∈ *S* of System (10) that satisfies Eq (17)

$$\begin{array}{c} J\left(E^{*}\right)=\underset{E\in S}{\sup}J\left(E\right). \end{array}$$ (17)

### 3.2 Solution of optimal strategy

In the following, we investigate the extremal Problem (9) using discrete time optimal control theory [26, 28]. To directly apply this theory, we should minimize the objective function. That is, solving Eq (9) is equivalent to solve the following equation

$$\begin{array}{c} \left(-J\right)\left(E\right)=\sum_{i=1}^{q}\left(LE_{i}-P\left(1-x\left(t_{i}\right)\right)E_{i}\right). \end{array}$$ (18)

Our main task is to find the optimal control E* ∈ *S*, which satisfies Eq (19)

$$\begin{array}{c} -\left(J\right)\left(E^{*}\right)=\underset{E\in S}{\inf}\left(-J\right)\left(E\right). \end{array}$$ (19)

Denote

$$\begin{array}{ccc} f_{0} & = & 0,\\ g_{0} & = & LE_{i}-P\left(1-x\left(t_{i}\right)\right)E_{i},\\ f_{1} & = & \frac{s\left(t\right)\eta_{1}\left(t\right)}{C}\left(1-x\left(t\right)\right)-\frac{x(t)}{C\eta_{2}\left(t\right)},\\ g_{1} & = & E_{i}\left(1-x\left(t_{i}\right)\right). \end{array}$$ (20)

We can gain the continuous Hamilton function *H* and the impulsive Hamilton function *H*_*c*_, respectively

$$\begin{array}{c} \left\{ \begin{array}{ccc} H & = & f_{0}+\lambda f_{1}\\ \; & = & \lambda (\frac{s\left(t\right)\eta_{1}\left(t\right)}{C}\left(1-x\left(t\right)\right)-\frac{x(t)}{C\eta_{2}\left(t\right)}),\\ H_{c} & = & g_{0}+\lambda \left(t_{i}^{+}\right)g_{1}\\ & = & \left(LE_{i}-P\left(1-x\left(t_{i}\right)\right)E_{i}\right)+\lambda \left(t_{i}^{+}\right)E_{i}\left(1-x\left(t_{i}\right)\right), \end{array}\right. \end{array}$$ (21)

where *λ* = *λ*(*t*) is the costate variable.

If $\left\{E_{i}^{*},i=1,2,\cdots ,q\right\}$ is the optimal control sequence and {x*(*t*_*i*_), *i* = 1, 2, ⋯, *q*} is the corresponding optimal trajectory, then there must exist a costate variable *λ* = *λ*(*t*) that satisfies the costate Eq (22)

$$\begin{array}{c} \left\{ \begin{array}{c} \frac{d\lambda }{dt}=-\frac{\partial H}{\partial x}=-\lambda \left(-\frac{s\left(t\right)\eta_{1}\left(t\right)}{C}-\frac{1}{C\eta_{2}\left(t\right)}\right)=\lambda F_{1}\left(t\right),t\neq t_{i},\\ \lambda \left(t_{i}\right)=\lambda \left(t_{i}^{+}\right)+\frac{\partial H_{c}}{\partial x}=\lambda \left(t_{i}^{+}\right)\left(1-E_{i}\right)+PE_{i},\;t=t_{i},\\ \lambda (t)=\lambda (t+T). \end{array}\right. \end{array}$$ (22)

Since *H*_*c*_ obtains its minimum value at the optimal control E*, we can know that E* satisfies the singular condition

$$\begin{array}{c} \frac{\partial H_{c}}{\partial E}=L-P\left(1-x\left(t_{i}\right)\right)+\lambda \left(t_{i}^{+}\right)\left(1-x\left(t_{i}\right)\right)=0. \end{array}$$ (23)

Using Eq (23), we get

$$\begin{array}{c} \lambda \left(t_{i}^{+}\right)=P-\frac{L}{1-x(t_{i})}. \end{array}$$ (24)

Integrating the first equation of Eq (22) from *t*_*i*_ to *t*_*i*+1_, we get

$$\begin{array}{c} \lambda \left(t_{i+1}\right)=\lambda \left(t_{i}^{+}\right)e^{\int_{t_{i}}^{t_{i+1}}F_{1}\left(s\right)ds}=\frac{\lambda (t_{i}^{+})}{D_{i+1}}. \end{array}$$ (25)

Substituting Eq (24) into Eq (25) yields

$$\begin{array}{c} \lambda \left(t_{i+1}\right)=\frac{1}{D_{i+1}}\left(P-\frac{L}{1-x(t_{i})}\right). \end{array}$$ (26)

Besides, substituting Eq (24) into the second equation of Eq (22) yields

$$\begin{array}{c} \lambda \left(t_{i+1}\right)=\left(1-E_{i+1}\right)\left(P-\frac{L}{1-x(t_{i+1})}\right)+PE_{i+1}. \end{array}$$ (27)

Combining Eq (26) with Eq (27) gives a set of relationships between the optimal solutions $E_{i}^{*}$ and x*(*t*_*i*_) (*i* = 1, 2, ⋯, *q*)

$$\begin{array}{c} \frac{1}{D_{i+1}}\left(P-\frac{L}{1-x(t_{i})}\right)=\left(1-E_{i+1}\right)\left(P-\frac{L}{1-x(t_{i+1})}\right)+PE_{i+1}. \end{array}$$ (28)

For another, the solution of the state Eq (10) with initial value $x\left(t_{0}^{+}\right)=x\left(0\right)$ can be solved as

$$\begin{array}{ccc} x(t) & = & x\left(0\right)\prod_{t_{0}<t_{i}<t}\left(1-E_{i}\right)e^{-\int_{t_{0}}^{t}F_{1}\left(s\right)ds}+\sum_{t_{0}<t_{i}<t}\left(\prod_{t_{i}<t_{j}<t}\left(1-E_{j}\right)e^{-\int_{t_{i}}^{t}F_{1}\left(s\right)ds}\right)E_{i}\\ & & +\int_{t_{0}}^{t}\prod_{s<t_{i}<t}\left(1-E_{i}\right)e^{-\int_{s}^{t}F_{1}\left(\sigma \right)d\sigma }F_{2}\left(s\right)ds. \end{array}$$ (29)

In particular, for *t* = *t*_*i*+1_ we have

$$\begin{array}{c} x\left(t_{i+1}\right)=x\left(t_{i}^{+}\right)e^{-\int_{t_{i}}^{t_{i+1}}F_{1}\left(s\right)ds}+\int_{t_{i}}^{t_{i+1}}e^{-\int_{s}^{t_{i+1}}F_{1}\left(\sigma \right)d\sigma }F_{2}\left(s\right)ds. \end{array}$$ (30)

For convenience, we denote

$$\begin{array}{ccc} B_{i+1} & = & \int_{t_{i}}^{t_{i+1}}e^{-\int_{s}^{t_{i+1}}F_{1}\left(\sigma \right)d\sigma }F_{2}\left(s\right)ds,\\ D_{i+1} & = & e^{-\int_{t_{i}}^{t_{i+1}}F_{1}\left(s\right)ds}. \end{array}$$ (31)

Then we can simplify Eq (30) as follows, called the stroboscopic map of System (10), which provides another set of relationships between the optimal solutions $E_{i}^{*}$ and x*(*t*_*i*_) (*i* = 1, 2, ⋯, *q*)

$$\begin{array}{c} x\left(t_{i+1}\right)=\left(\left(1-E_{i}\right)x\left(t_{i}\right)+E_{i}\right)D_{i+1}+B_{i+1}. \end{array}$$ (32)

Due to the periodical condition for any *i*, we know *x*_*i*+*q*_ = *x*_*i*_ and *E*_*i*+*q*_ = *E*_*i*_. We can acquire 2*q* equations which comprise 2*q* unknown variable vectors *E*_*i*_ and *x*(*t*_*i*_) by setting *i* = 1, 2, ⋯, *q* in Eqs (28) and (32). These equations constitute the optimization system of the optimal control Problem (9). Consequently, we can get the optimal teaching strategy {E*, *i* = 1, 2, ⋯, *q*} and the corresponding optimal knowledge level {x*(*t*_*i*_), *i* = 1, 2, ⋯, *q*} through this system by numerical methods. Further the maximum learning effectiveness in a period can be got through the expression of *J*.

## Results

We provide several practical examples in this section. We firstly analyze *q* = 1 theoretically, namely, only one teaching activity occurring at the fixed moment per period. Under certain conditions, the optimal control strategy can be completely determined in this case.

We denote

$$\begin{array}{ccc} E & = & E_{1},x=x\left(t_{1}\right),\\ D & = & e^{-\int_{0}^{T}F_{1}\left(s\right)ds},\\ B & = & \int_{0}^{T}e^{-\int_{s}^{T}F_{1}\left(\sigma \right)d\sigma }F_{2}\left(s\right)ds. \end{array}$$ (33)

Then

$$\begin{array}{c} J(E)=(P(1-x)-L)E. \end{array}$$ (34)

On the basis of Eq (33), it follows from Eq (32) that

$$\begin{array}{c} x=\frac{B+DE}{1-D+DE}. \end{array}$$ (35)

Substituting Eq (35) into Eq (28), one has

$$\begin{array}{c} {\left(1-D+DE\right)}^{2}=\frac{P}{L}\left(D-1\right)\left(B+D-1\right):=A. \end{array}$$ (36)

Therefore, if *B* + *D* ≤ 1 holds, then *E* can be solved from Eq (36) as

$$\begin{array}{c} E=\frac{\sqrt{A}+D-1}{D}. \end{array}$$ (37)

Meanwhile, substituting Eq (37) into Eq (35) yields

$$\begin{array}{c} x=\frac{\sqrt{A}+B+D-1}{\sqrt{A}}. \end{array}$$ (38)

The solutions *E* and *x* are in the interval from zero inclusive to one exclusive when (1 − *D*)^2^ ≤ *A* < 1 holds. In this manner, we can conclude that the optimal solutions E* and x* are uniquely determined and given by Eqs (37) and (38), with the conditions *B* + *D* ≤ 1 and (1 − *D*)^2^ ≤ *A* < 1 holding together.

Furthermore, the maximum learning effectiveness in a period can be obtained through Eq (39)

$$\begin{array}{c} J^{*}=\frac{\left(P\left(1-B-D\right)-L\sqrt{A}\right)\left(\sqrt{A}+D-1\right)}{\sqrt{A}D}. \end{array}$$ (39)

Next, we numerically analyze *q* in other cases. We know different learners possess diverse benefits and costs in the same knowledge dissemination process. The benefits and costs raised by different process are also unlike toward the same learner. Hence, to begin with we can work out the teaching plan (the times and the intervals of impulsive teaching activity per period) and ascertain the learning style (the capability of a learner to absorb knowledge and retain memory). Further we need to make sure the benefits and costs aimed at the particular learner in the specific knowledge dissemination process. Then we can numerically solve the optimization system constituted by Eqs (28) and (32) to obtain the optimal teaching strategy and the optimal knowledge level step by step in Maple.

Specifically, we numerically solve the optimal solutions under three different teaching plans (*q* = 1, *q* = 2 and *q* = 3). For functions *s*(*t*), *η*_1_(*t*) and *η*_2_(*t*), we select one as the periodic function, whereas the others are assigned as the constant functions. This setting is to make an analogy to diverse learning styles [29], as shown in Table 1, which aims to exhibit the universality of the optimal teaching strategy.

**Table 1:** Diverse learning styles.

Styles	Corresponding periodic functions *s*(*t*), *η*_1_(*t*) and *η*_2_(*t*)
1	$s\left(t\right)=\frac{1}{2}\left(\tanh\left(\sin\frac{\pi t}{5}\right)+1\right),\;\eta_{1}\left(t\right)=0.6,\;\eta_{2}\left(t\right)=0.6$
2	$s\left(t\right)=1,\;\eta_{1}\left(t\right)=\frac{1}{2}\left(\cos\frac{\pi t}{5}+1\right),\;\eta_{2}\left(t\right)=0.6$
3	$s\left(t\right)=1,\;\eta_{1}\left(t\right)=0.6,\;\eta_{2}\left(t\right)=\frac{1}{2}\left(\sin\frac{\pi t}{5}+1\right)$

We select the following parameters to calculate: *α* = 1, *β* = 1, *C* = 10, *t*_1_ = 1, *t*_2_ = 3, *t*_3_ = 5, *T*_*m*_ = 5, *T* = 10, *P* = 5 and *L* = 1. We assume that only one impulsive teaching activity takes at the fixed moments *t*_1_ = 1 per period *T* = 10 when *q* = 1. Similarly, we conduct two impulsive teaching activities at *t*_1_ = 1, *t*_2_ = 3 when *q* = 2 and three activities at *t*_1_ = 1, *t*_2_ = 3, *t*_3_ = 5 when *q* = 3 within the same period. *E*_*i*_(*i* = 1, 2, 3) are their corresponding impulsive teaching effort. According to the above method, we can get the results as shown in Table 2. Each row displays the optimal solutions in the corresponding situations.

**Table 2:** Numerical optimal solutions in corresponding situations.

Teaching Plans	Styles	E* = (*E*_1_, ⋯, *E*_*q*_)	x* = (*x*_1_, ⋯, *x*_*q*_)	J*
*q* = 1	1	0.2309	0.5973	0.2340
	2	0.2349	0.6441	0.1830
	3	0.2167	0.6203	0.1947
*q* = 2	1	(0.2847, 0.3593)	(0.6472, 0.6123)	0.5547
	2	(0.2943, 0.3315)	(0.6700, 0.6430)	0.4516
	3	(0.1741, 0.2732)	(0.6600, 0.6586)	0.3150
*q* = 3	1	(0.3003, 0.2110, 0.4423)	(0.6828, 0.6319, 0.6329)	0.7227
	2	(0.3420, 0.4730, 0.2872)	(0.6394, 0.6402, 0.6094)	0.9261
	3	(0.1442, 0.1483, 0.3392)	(0.6953, 0.6730, 0.6909)	0.3547

The table depicts that, for teachers, the results provide a quantitative basis to make their teaching strategies pertinently and designedly. Naturally, learners can gain maximum benefits at minimum costs. Therefore, faced with the complex and complicated personal education, our research can fulfill various teaching and learning requirements, thereby showing its superiority.

## Conclusion and discussions

In this paper, we propose the periodic impulsive knowledge dissemination system, which is more accordant with the laws of knowledge dissemination affected by the teaching activities. This system reflects that the learning and progress of a learner can not be separated from the teacher guidance. Therefore, it is crucial to draw up the suitable teaching strategy that complies with the requirements of both teachers and learners. Such teaching strategy needs to be measurable and operable, and not simply ubiquitous and qualitative descriptions.

Our study through strict mathematical derivation and analysis does not only exhibit intrinsic stability, but also can solve this problem properly. Meanwhile, we give several practical examples to make the conclusion intuitive and specific. Certainly, the more delicately the learning styles of learners are portrayed, the more complicated the optimization system is solved. We need to use some powerful mathematical tools to complete the calculation that can not be completed manually. Clearly, this quantitative study is also applicable for open online learning and e-learning by addressing the problem of assigning the most suitable capacity of learning materials at specified times for learners.

In the future, we can select impulsive moments as control variables (assuming *E*_*i*_, *i* = 1, 2, ⋯, *q* are fixed), and propose other management objective, such as average knowledge absorptive capacity [30, 31] within a period. Investigating which sequences of impulsive moments can maximize objective function is also a meaningful work. The findings can cope with the problem of identifying the most appropriate series of times to send certain learning materials to learners. Research on the above two kinds of problems can realize the functions of pushing learning materials toward learners quantitatively and regularly in open online learning and e-learning.

In conclusion, realizing quantitative description and solution for actual changes and thorough processes of knowledge dissemination is a fundamental task crucial for precisely drawing up the efficient teaching strategy. Such customized strategy is beneficial and practical because it considers the development requirements of learners, provides quantitative basis for teaching process, and highlights the advantages of personalized education. We believe that we can create an improved learning environment for learners by optimizing teaching strategy to appeal to a wide variety of learning styles.
